# Deprescribing practices, habits and attitudes of geriatricians and geriatricians-in-training across Europe: a large web-based survey

**DOI:** 10.1007/s41999-022-00702-9

**Published:** 2022-11-02

**Authors:** Eveline P. van Poelgeest, Lotta J. Seppala, Jihoo M. Lee, Gülistan Bahat, Birkan Ilhan, Amanda H. Lavan, Alpana Mair, Rob J. van Marum, Graziano Onder, Jesper Ryg, Marília Andreia Fernandes, Doron Garfinkel, Aðalsteinn Guðmundsson, Sirpa Hartikainen, Marina Kotsani, Beatriz Montero-Errasquín, Agnieszka Neumann-Podczaska, Farhad Pazan, Mirko Petrovic, George Soulis, Hana Vankova, Martin Wehling, Katarzyna Wieczorowska–Tobis, Nathalie van der Velde, On Behalf of the EuGMS SIG Pharmacology

**Affiliations:** 1 Department of Internal Medicine Section of Geriatrics, Amsterdam University Medical Centers, Location University of Amsterdam, Meibergdreef 9, Amsterdam, The Netherlands; 2 Amsterdam Public Health Research Institute, Aging and Later Life, Amsterdam, The Netherlands; 3 Graduate School of Communication Science, University of Amsterdam, Amsterdam, The Netherlands; 4 Division of Geriatrics, Department of Internal Medicine, Istanbul Medical School, Istanbul University, Capa, Istanbul, Turkey; 5 Division of Geriatrics, Department of Internal Medicine, Sisli Hamidiye Etfal Training and Research Hospital, University of Medical Sciences, Istanbul, Turkey; 6 Mercers Institute of Successful Ageing, St James’s Hospital, Dublin, Ireland; 7 Effective Prescribing and Therapeutics, Health and Social Care Directorate, Scottish Government, Edinburgh, Scotland UK; 8 Department of Elderly Care Medicine, Amsterdam University Medical Centers, Location Vrije Universiteit Amsterdam, De Boelelaan 1117, Amsterdam, Netherlands; 9 Department of Cardiovascular, Endocrine-Metabolic Diseases and Aging, Istituto Superiore di Sanità, Rome, Italy; 10 Department of Geriatric Medicine, Odense University Hospital, Odense, Denmark; 11 Department of Internal Medicine, Hospital Curry Cabral, Centro Hospitalar Universitário de Lisboa Central, Lisbon, Portugal; 12 Center for Appropriate Medication Use, Sheba Medical Center and Deputy Head, Homecare Hospice, Israel Cancer Association, 55 Ben Gurion Road, 5932210 Bat, Yam Israel; 13 Landspitali University Hospital, Reykjavik, Iceland; 14 School of Pharmacy, University of Eastern Finland, Kuopio, Finland; 15 Université de Lorraine, CHRU-Nancy, Pôle (Maladies du Vieillissement, Gérontologie et Soins Palliatifs), Nancy, France; 16 Servicio de Geriatría, Hospital Universitario Ramón y Cajal, IRYCIS, Madrid, Spain; 17 Geriatrics Unit, Department of Palliative Medicine, Poznan University of Medical Sciences, Poznan, Poland; 18 Clinical Pharmacology Mannheim, Medical Faculty Mannheim, Heidelberg University, Mannheim, Germany; 19 Section of Geriatrics, Department of Internal Medicine and Paediatrics, Ghent University, Ghent, Belgium; 20 Outpatient Geriatric Assessment Unit, Henry Dunant Hospital Center, Athens, Greece; 21 Cooperatio 34 - Internal Disciplines, Third Faculty of Medicine, Charles University, Prague, Czech Republic; 22 Department of Medical Gerontology, Trinity College Dublin, Dublin, Ireland; 23 Geriatric Research Unit, Department of Clinical Research, University of Southern Denmark, Odense, Denmark; 24 Odense Deprescribing Initiative (ODIN), Odense University Hospital and University of Southern Denmark, Odense, Denmark; 25 Faculty of Medicine, University of Iceland, Reykjavik, Iceland; 26 Hellenic Open University, Patras, Greece

**Keywords:** Deprescribing, Geriatric medicine, Older adults, Adverse drug effects, Online survey, Medication review

## Abstract

**Purpose:**

To provide an overview of the current deprescribing attitudes, practices, and approaches of geriatricians and geriatricians-in-training across Europe.

**Methods:**

An online survey was disseminated among European geriatricians and geriatricians-in-training. The survey comprised Likert scale and multiple-choice questions on deprescribing approaches and practices, deprescribing education and knowledge, and facilitators/barriers of deprescribing. Responses to the survey questions and participant characteristics were quantified and differences evaluated between geriatricians and geriatricians-in-training and between European regions.

**Results:**

The 964 respondents (median age 42 years old; 64% female; 21% geriatricians-in-training) were generally willing to deprescribe (98%) and felt confident about deprescribing (85%). Despite differences across European regions, the most commonly reported reasons for deprescribing were functional impairment and occurrence of adverse drug reactions. The most important barriers for deprescribing were patients’ unwillingness, fear of negative consequences, lack of time, and poor communication between multiple prescribers. Perceived risk of adverse drug reactions was highest for psychotropic drugs, nonsteroidal anti-inflammatory drugs, cardiovascular drugs, and opioid analgesics. Only one in four respondents (23% of geriatricians and 37% of geriatricians-in-training) think education in medical school had sufficiently prepared them for deprescribing in clinical practice. They reported that their future deprescribing activities would probably increase with improved information sharing between various prescribers, deprescribing recommendations in guidelines, and increased education and training. Approximately 90% think that a paradigm shift is required for prescribers and patients, increasing focus on the possible benefits of deprescribing (potentially) inappropriate medications.

**Conclusions:**

Based on the outcomes of this survey, we recommend investing in improved inter-professional communication, better education and evidence-based recommendations to improve future patient-centered deprescribing practices.

**Supplementary Information:**

The online version contains supplementary material available at 10.1007/s41999-022-00702-9.

## Key summary points

**Aim:**

To explore current deprescribing attitudes and practices among geriatricians and geriatricians-in-training across Europe.

**Findings:**

Functional decline, adverse drug reactions and adherence to guidelines/checklists for managing polypharmacy were the most important reasons for deprescribing. The most important barriers for deprescribing were patients’ unwillingness, fear of negative consequences, lack of time, and poor communication between multiple prescribers. Only one in four respondents thought education in medical school had sufficiently prepared them for deprescribing in clinical practice.

**Message:**

There is a need for improved inter-professional communication, better education and evidence-based recommendations to improve future person-centered prescribing and resultant deprescribing practices.

**Supplementary Information:**

The online version contains supplementary material available at 10.1007/s41999-022-00702-9.

## Introduction

Over the past decades, the number of medications prescribed to older adults has increased considerably [1]. In Western societies, over 50% of community-dwelling older adults (aged ≥ 65 years old) are exposed to polypharmacy [2–4], usually defined as the chronic use of five or more medications [5, 6]. Besides the potential positive health effects of these medications, polypharmacy also increases the risk of potentially inappropriate medications (PIMs) [7]. Inappropriate medication use is highly prevalent in older adults: up to 40% of medications prescribed to older adults in primary care is potentially inappropriately prescribed [8, 9]. PIMs contribute to negative medical, economic, and social consequences, such as adverse drug events, drug-related hospitalizations, high health care costs, and mortality [7, 10–13].

In this context, structured medication review and optimization have emerged as a potentially effective strategy to improve patient outcomes [14] and reduce inappropriate polypharmacy [15, 16], medication-related adverse events [17] and healthcare-related costs [18]. Deprescribing is the process of withdrawal of an inappropriate medication, supervised by a healthcare professional with the goal of managing polypharmacy and improving outcomes [19]. Furthermore, polypharmacy management is currently a worldwide top priority in patient safety and is highlighted in the flagship report "medication safety in polypharmacy" [20] as a priority area to address. According to the World Health Organization (WHO) Third Global Patient Safety Challenge ‘Medication without Harm’ in 2017, the aim is to globally reduce severe avoidable medication-related harm by 50% over 5 years [21]. However, some studies suggest that deprescribing is not routinely performed in everyday clinical practice [11, 22]. In addition, deprescribing practices appear to vary considerably within and between countries and regions in Europe [10]. (De)prescribing medications is an important component of geriatricians’ daily practice because they treat predominantly multimorbid older adults with polypharmacy, who are most likely to have PIMs. Understanding the deprescribing attitudes of geriatricians, the barriers they face, and the potential differences between countries is paramount in identifying the key areas which, when addressed could result in improved deprescribing practices across Europe. Therefore, the aims of this project were to provide an overview of the current deprescribing attitudes, practices, and approaches of geriatricians and geriatricians-in-training to deprescribing across Europe, and to explore potential regional differences herein.

## Methods

### Design

This cross-sectional web-based survey was initiated and conducted by members of the European Geriatric Medicine Society (EuGMS) Special Interest Group (SIG) on Pharmacology and coordinated by investigators from the University of Amsterdam, The Netherlands. The steering (core) committee of the project consisted of international experts with experience and knowledge in the field of geriatric medicine, geriatric pharmacotherapy, and/or communication science. The original questionnaire was created by first conducting a scoping literature review on all processes involved in patient-centered deprescribing. Data extracted were discussed by the steering committee. After selecting the items to include in the survey, the survey questions were drafted and linguistically checked by native English speakers of the steering committee.

Prior to dissemination of the questionnaire to potential participants, the questionnaire was piloted by the steering committee and five independent Dutch geriatricians and geriatricians-in-training to evaluate its clarity, feasibility, and amount of time required for completion. Based on the suggestions of the piloting panel, the questionnaire was adapted. National representatives (members of the EuGMS SIG on Pharmacology) from 20 countries collaborated on the project. The representatives were involved in translating the questionnaire into their national languages (required for Poland, Germany, Finland, Spain, Austria, Czech Republic, France, Italy, and Belgium) and promoting participation among their national colleagues. All (translated) versions of the questionnaire had a unique URL embedded in and delivered via an email invitation to the members of national geriatric societies. In countries where it was not possible to use email lists for privacy reasons (e.g. Germany and The Netherlands), participation was encouraged by notifications in newsletters and on websites. In addition, personal networks of the investigators were notified. After the initial distribution, a maximum of two reminder emails was sent. Survey participation was also encouraged by the EuGMS through an invitational email sent to all its members, a banner on its official website, and social media channels. The survey was conducted from June 19th to November 22nd, 2021.

### Study population

Eligible participants were European geriatricians and geriatricians-in-training. For those countries formally lacking geriatric medicine as a specialty (Greece and Portugal), hospital-based physicians specializing in care of older patients (and their trainees) were eligible, and are further in this paper referred to as geriatricians. Based on the response rate among European physicians treating older adults in an earlier survey project [23], and the fact that this would be sufficient to perform sub analyses, we aimed to include approximately 1000 participants from as many European countries as possible. No formal power calculation was performed.

### Ethics

The study protocol and survey questionnaire were sent to the ethics committee of the Amsterdam Medical Center, University of Amsterdam, who concluded that no ethical approval was required for this survey study among physicians (W21_145 # 21.160). For Ireland, Spain, Turkey, Poland, and Belgium, formal approval from their respective ethical committees was necessary and granted. Prior to entering the survey questions, digital informed consent was obtained from all participants. Collected data were uploaded from the LimeSurvey platform to an SPSS database on the internal research IT-infrastructure of the projects research team (located at the Amsterdam University Medical Centers, The Netherlands), protected against unauthorized access. Participation was voluntary, participant data were anonymized, and participants could withdraw at any time without any consequence of any kind.

### Data collection and questionnaire

The final survey instrument (Supplementary material) was uploaded to the LimeSurvey open-source online questionnaire program (Version 2.6.7 Build SondagesPro 1.7.3.) and contained 20 questions divided into four main domains: (1) participants’ characteristics; (2) deprescribing approaches and practices; (3) deprescribing education and knowledge; and (4) facilitators/barriers of deprescribing. The majority of questions were multiple-choice questions with Likert scale answer options.

We categorized the county of the participants in European regions according to the geographical definition of the United Nations (based on homogeneity in economic or social factors; https://unstats.un.org/unsd/methodology/m49/). Turkey and Israel were categorized as Eastern Europe, and Cyprus as Southern Europe, based on their geographical location. The Eastern European region contained Belarus, Bulgaria, Czech Republic, Israel, Poland, Romania, and Turkey; the Northern European region Denmark, Finland, Iceland, Ireland, Lithuania, Norway, Sweden, and United Kingdom; the Southern European region Cyprus, Greece, Italy, Republic of North Macedonia, Malta, Portugal, Serbia, Slovenia, and Spain; the Western European region Austria, Belgium, France, Germany, Luxembourg, Switzerland, and The Netherlands.

To reduce the number and complexity of questions, we used conditional questioning. Participants were encouraged to complete the questionnaire by notifications on each page. Notifications pointed out which answers were not filled in (proceeding to the next page was only possible after filling in all answers to the previous page). Open text answers were translated into English language using an automated translation program and categorized for analysis. In case of uncertainty, the native speaking national representatives were contacted.

### Statistical analysis

The participants’ characteristics data were both calculated for all participants and categorized according to European region. We calculated frequencies for categorical variables and means with standard deviations or medians with interquartile ranges for continuous variables. In addition, we reported the participant characteristics data for certified geriatricians and geriatricians-in-training.

We quantified the perceived clinical importance of 12 potential deprescribing barriers by calculating Quality Impact Indices [24] for a number of factors that participants deem challenging when deprescribing. To that end, the mean score of the 4-point Likert scale to the statement “I find deprescribing challenging due to …” (1 = not challenging; 2 = a little challenging; 3 = challenging; 4 = extremely challenging) was multiplied by the fraction of participants that selected “yes” to the question “In the past month, have you been reluctant to deprescribe due to …. (the respective factor)” [24].

For analysis of the frequency of generally performing the 5 steps of the patient-centered deprescribing process as described by Reeve et al. [25] we calculated the number of participants who selected “often” or “always” on the 4-point Likert scale, ranging from “never” to “always”. We analyzed differences between regions and trainees and geriatricians using the chi-square test. For the remainder of the survey items, we calculated distributions of Likert scales and frequencies (for example to identify the most frequently reported enablers for deprescribing). We used a threshold of *p* < 0.05 for statistical significance. All statistical analyses were performed in IBM SPSS Statistics for Windows, Version 26.0 (IBM Corp, NY).

## Results

### Participant characteristics

In total, 964 physicians (79% geriatricians and 21% geriatricians-in-training; Table 1) from 31 countries participated, with 73% completing all questions asked. The number of participants per country ranged from one to 127 (Supplementary Table 1). Most participants from the Eastern, Northern, Southern and Western European region were from Turkey and Czech Republic, Denmark and United Kingdom, Italy and Spain, and France and The Netherlands, respectively. Median age of the participants was 42 (IQR 35, 54) years and 64% were female (Table 1). The median number of years of experience as a doctor in geriatric medicine for all participants was 10 (IQR 5, 20; for geriatricians median 13 years, and for geriatricians-in-training 3 years). Fifty-four percent of the participants had internal medicine, 15% palliative care, and 4% clinical pharmacology as a (sub) specialty interest.

**Table 1:** Characteristics of participants in deprescribing survey per European region

	Total (*n* = 964)	Eastern Europe (*n* = 162)	Northern Europe (*n* = 259)	Southern Europe (263)	Western Europe (*n* = 280)
Age (years; median (IQR))	42 (35,54)	40 (34,50)	44 (37,55)	44 (33,55)	40 (34,53)
Gender (% female)	64	70	67	58	64
Trainee (% yes)	21	29	21	18	19
Experience as medical doctor (years, median (IQR))	15 (8,26)	15 (9,26)	17 (11,28)	16 (7,28)	13 (7,25)
Experience in geriatric medicine (years; median (IQR))	10 (5,20)	8 (3,15)	10 (6,18)	13 (5,25)	9 (4,17)
Deprescribing activities (mean % of total consultations)	59	54	60	63	58
Subspecialty besides geriatric medicine (% yes)	40	48	44	24	45
General medicine	15	9	17	27	13
Internal or geriatric medicine subspecialty	54	76	64	43	36
Clinical pharmacology	4	0	3	2	8
Palliative care	15	13	10	10	25
Other	12	3	7	19	18
Current health care setting (%)					
Community	6	8	10	7	1
Outpatient clinic	19	35	15	13	19
Clinical ward	59	44	64	57	64
Long term care or rehabilitation setting	13	11	7	20	13
Other	3	3	5	4	2

*IQR* interquartile range

Eastern Europe: Belarus, Bulgaria, Czech Republic, Israel, Poland, Romania and Turkey; Northern Europe: Denmark, Finland, Iceland, Ireland, Lithuania, Norway, Sweden and United Kingdom; Southern Europe: Cyprus, Greece, Italy, Macedonia, Malta, Portugal, Serbia, Slovenia and Spain; Western Europe: Austria, Belgium, France, Germany, Luxembourg, Switzerland and The Netherlands

### Deprescribing approaches and practices

**Fig. 1:**
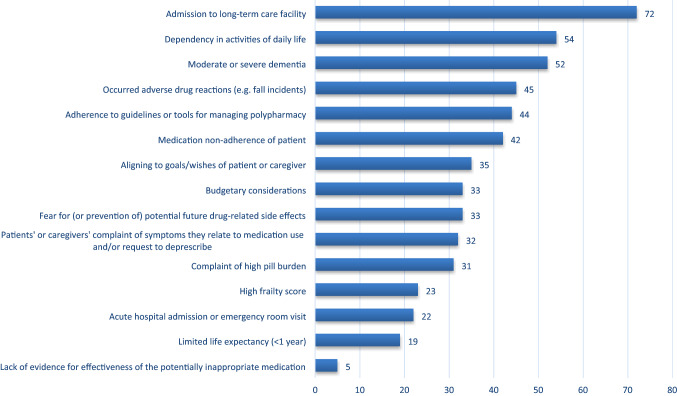
Most important reasons for deprescribing according to European geriatricians and geriatricians-in-training (percentage of participants selecting the respective reasons in their Top 5 selection)

**Fig. 2:**
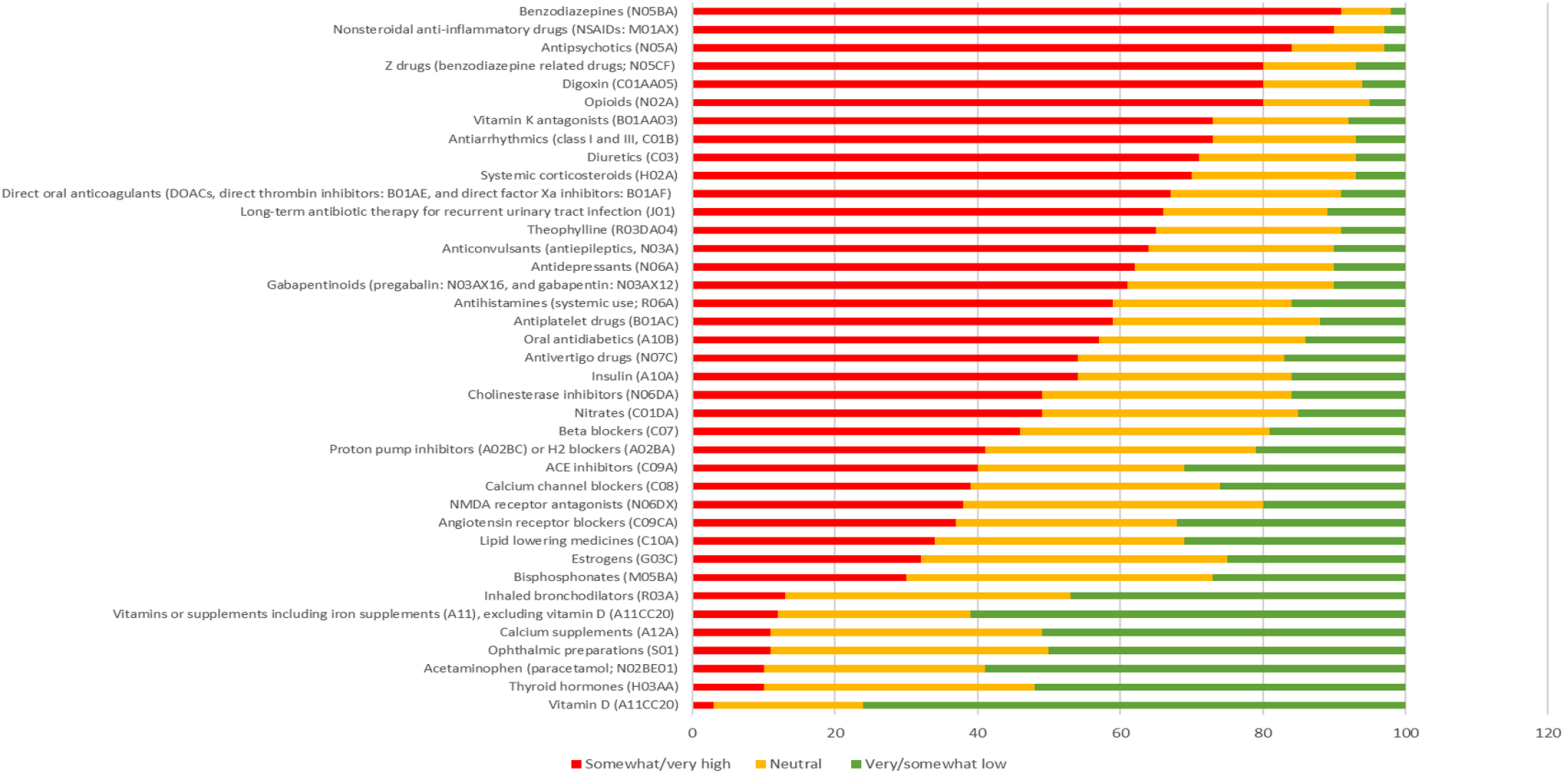
Distributions of level of agreement with the statement “In multimorbid older adults, I perceive the risk of adverse drug reactions related to this medication (group) as …” on a 5-point Likert scale (“very high” to “very low”) among 964 European geriatricians and geriatricians-in-training

Participants estimated that they deprescribe medications they consider (potentially) inappropriate in 59% of patient consultations. Eighty-five percent felt confident about deprescribing, almost all (98%) participants reported a general willingness to deprescribe, and 97% reported to be pro-active in deprescribing. The five most reported reasons for deprescribing (Fig. 1) were admission to a long-term care facility (72%, *n* = 572), dependency in activities of daily living (54%, *n* = 431), moderate/severe dementia (52%, *n* = 412), adverse drug reaction occurrence e.g. fall incidents (45%, *n* = 358), and adherence to guidelines or tools for managing polypharmacy (44%, *n* = 352). Almost all (97%) participants reported to take the perceived risk of adverse drug reactions into account when deprescribing. Antipsychotics, benzodiazepines, Z-drugs (e.g. zopiclone and zolpidem), non-steroidal anti-inflammatory drugs (NSAIDs) and opioid analgesics are in the top 5 drugs with the highest perceived risk of adverse events in multimorbid older adults (Fig. 2).

The five most used explicit inappropriate polypharmacy and deprescribing checklists and online resources (Supplementary Fig. 1) were STOPP (Screening Tool of Older Person’s Prescriptions) criteria [26], Beers criteria [27], STOPPFrail [28], STOPPFall [29], and deprescribing.org (https://deprescribing.org). Of the 20 participating countries for which a national representative collaborated in the project, comprehensive national guidelines for polypharmacy and/or deprescribing were available in the Czech Republic, Denmark, France, Ireland, Italy, Scotland, Sweden, and The Netherlands.

**Fig. 3:**
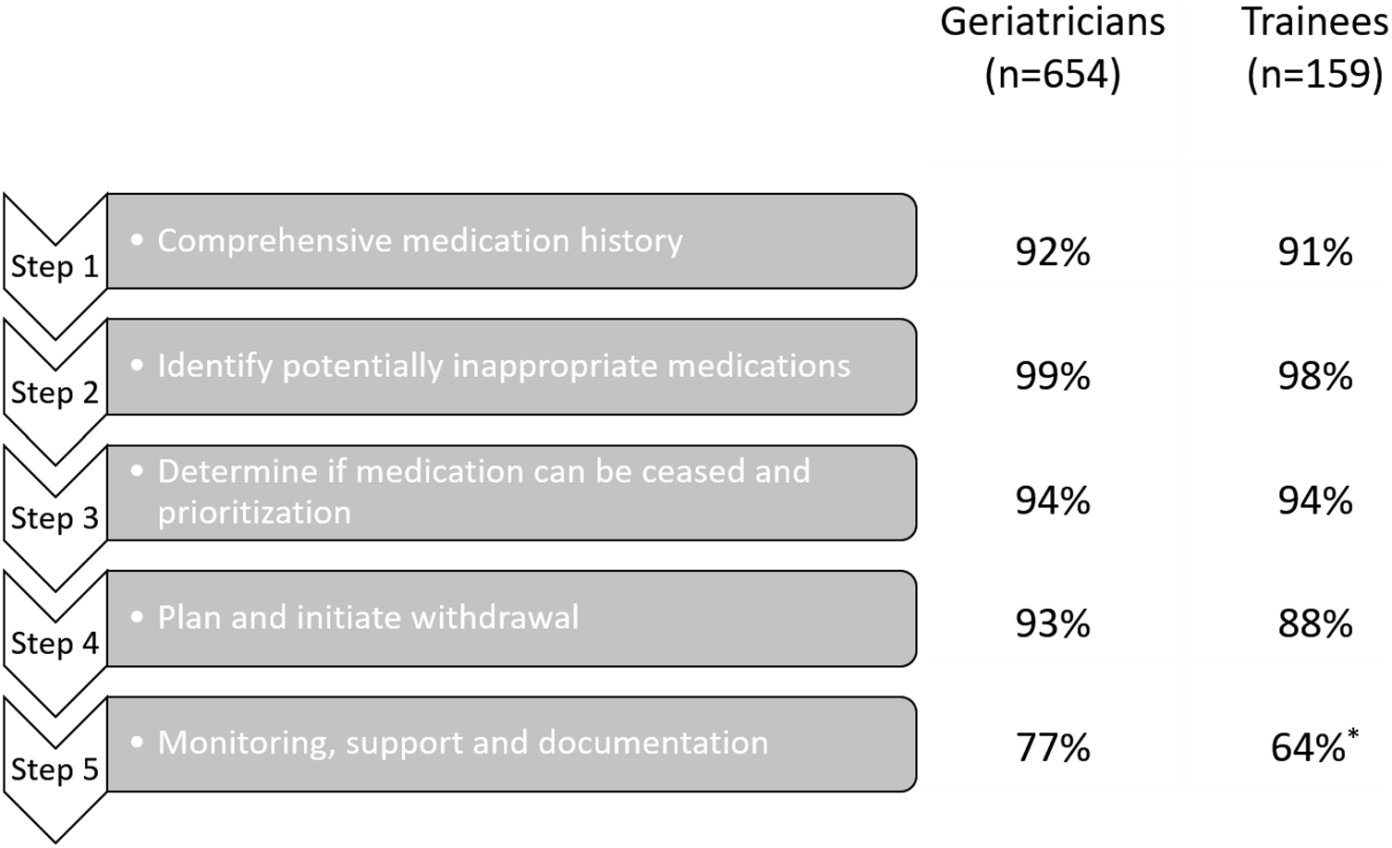
Percentage of geriatricians and geriatricians-in-training reporting to perform the five steps of the patient centered deprescribing process (Reeve et al. BJCP [25])

The majority (92–99%) of geriatricians and geriatricians-in-training reported to generally perform the first four steps of the patient-centered deprescribing process [25] (Fig. 3), whereas the final step (monitoring, support, and documentation) was generally performed by only 77% of geriatricians and 64% of geriatricians-in-training. Twenty-six percent of participants reported deprescribing medications initiated by another treating medical specialist without prior inter-specialty discussion; 65% informed and consulted the other specialist first. Forty-seven percent of participants reported to generally collaborate with the general practitioner (GP) when deprescribing, 29% with a pharmacist or clinical pharmacologist, and 42% with other medical specialists. Only 15% of participants reported to generally collaborate with specialized nurses when deprescribing.

Ninety-two percent of respondents reported to generally discuss deprescribing options with their patients and/or caregivers. The majority (94%) of respondents reported taking patient and/or caregiver preferences and treatment goals into account when deprescribing, 88% reported that they generally succeeded in motivating patients and/or caregivers to deprescribe (88%). Ninety-three percent of all respondents reported to think a culture change is required for prescribers (94% of geriatricians, and 91% of geriatricians-in-training; *p* = 0.33), with an increasing focus on the possible benefits of deprescribing (potentially) inappropriate medications. Eighty-nine percent think such a culture change is required for patients (90% of geriatricians, and 86% of geriatricians-in-training; *p* = 0.03).

### Deprescribing education and knowledge

Seventy-two percent of participants reported to have received education or training in reviewing polypharmacy and/or deprescribing (Supplementary Table 2). Compared to geriatricians, geriatricians-in-training reported more frequently to have been trained and educated in medical school (47 vs 23%; *p* < 0.05) and during residency and/or fellowship (83 vs 54%; *p* < 0.05). Geriatricians reported more often to have received training from conferences or meetings (75 vs 53%, *p* < 0.05). Twenty-six percent of all participants thought that education in medical school had prepared them adequately for deprescribing in clinical practice (23% of geriatricians, and 37% of geriatricians-in-training; *p* < 0.05). Seventy percent of all participants reported their general knowledge on deprescribing was good (72% of geriatricians, and 58% of geriatricians-in-training; *p* < 0.05), and 77% reported that their knowledge on which drugs require tapering was good (80% of geriatricians, and 59% of geriatricians-in-training; *p* < 0.05).

### Barriers and facilitators of deprescribing

**Fig. 4:**
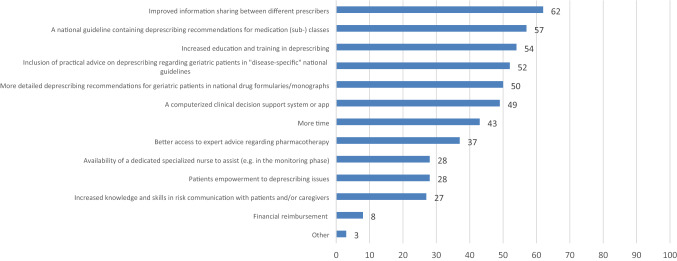
Factors European geriatricians and geriatricians-in-training think would most likely increase their future deprescribing activities (percentages)

The highest-ranked barriers to deprescribing were “Fear of potential negative patient health outcomes after deprescribing”, “Unwillingness to have medication dose lowered or withdrawn by patient and/or caregiver”, “Poor information sharing among patients' multiple prescribers”, and “Lack of time” (Supplementary Fig. 2). According to the participants (geriatricians and geriatricians-in-training alike), the most reported factors that would most likely increase their future deprescribing activities (Fig. 4) were “Improved information sharing between different prescribers”, “A national guideline containing deprescribing recommendations for medication (sub-) classes”, “Increased education and training in deprescribing”, and “Inclusion of practical advice on deprescribing for geriatric patients in disease-specific guidelines”. Results were comparable for geriatricians and geriatricians-in-training.

### Regional similarities and differences

The European regions differed in terms of percentage of participants reporting to be proactive in reviewing medications and considering deprescribing PIMs in their patients (East 92%, South 97%, West 98%, North 99%; *p* = 0.003), and feeling confident in deprescribing (South 75%, West 85%, East 88%, North 92%; *p* < 0.001). There were marked regional differences in the highest-ranked reasons for deprescribing across the European regions (Supplementary Table 2). The medicines (or medication classes) with highest perceived risk of adverse drug effects were comparable between the European regions (Supplementary Table 3).

The European regions also differed in the percentage of participants reporting to have received education or training in reviewing polypharmacy and/or deprescribing in medical school (South 6%, West 17%, North 23%, East 28%; *p* < 0.001), and in residency and/or fellowship in geriatric medicine (South and West 30%, East 40%, North 43%; *p* = 0.003).

Three items were in the top 5 of highest ranked barriers for deprescribing in all four European regions (fear of potential negative patient health outcomes after deprescribing, poor information sharing among patients’ multiple prescribers, and unwillingness to have medication dose lowered of drug withdrawn by patient and/or caregiver; Supplementary Table 4). In all four European regions, the highest ranked items participants thought would be most helpful to improve future deprescribing activities included detailed deprescribing recommendations in “disease-specific” national guidelines, national drug formularies/monographs, or in a national deprescribing guideline (Supplementary Fig. 3). However, overall, the highest-ranked items varied between the regions. For example, among the top 5 items only for participants from the Northern European region was more time (selected by 92% of participants), whereas better access to expert advice regarding pharmacotherapy was top-ranked only by participants from the Eastern European region (selected by 44% of participants).

## Discussion

In this study, we characterized deprescribing habits, attitudes, facilitators, barriers, and needs among European geriatricians and geriatricians-in-training. The findings showed that geriatricians are generally willing to deprescribe in older, multimorbid adults, and feel confident about performing all five recommended steps of patient-centered deprescribing. However, geriatricians relatively infrequent perform the last step of developing and implementing a deprescribing plan. Furthermore, geriatricians believed that their future deprescribing activities would increase with improved information sharing between various prescribers, deprescribing recommendations in guidelines, and increased education and training. The most important barriers for deprescribing were fear of negative consequences, patients’ unwillingness to deprescribing, lack of time, and poor communication between multiple prescribers. Only one in four participants thought education in medical school had prepared them for deprescribing in clinical practice, but there was a marked difference between geriatricians-in-training and geriatricians. Thirty-seven percent of geriatricians-in-training vs 23% of geriatricians thought that education had prepared them adequately.

Literature shows that developing and implementing a deprescribing patient-tailored plan is crucial for successful and sustainable deprescribing [30]. Yet, based on our findings, geriatricians and geriatricians-in-training relatively infrequently perform this last step. The fact that geriatricians mostly work on clinical wards, could contribute to this finding: after discharge, GPs instead of geriatricians may be in the lead in patient management. Furthermore, poor communication between different healthcare professionals [31] and the time-consuming nature of gathering information warranted for the plan could contribute to this finding.

Poor communication and/or information sharing was among the most important barriers to deprescribing in our study, and improved information sharing was one of the items that geriatricians think would increase their future deprescribing activities. Poor information sharing between different prescribers in geriatric patients has been well documented in previous literature, and expressed not only by geriatricians [32], but also by GPs [33, 34] and pharmacists [32]. Although often not readily available, detailed medication-related information (for example regarding the patients’ previous deprescribing attempts) is essential for optimal decision-making. Thus, improving information sharing (for example through shared health record systems) between different prescribers and across different healthcare settings may be a promising approach to optimize future safe deprescribing efforts.

Alternatively, development and implementation of a patient-centered deprescribing plan may be challenging for geriatricians because education and training in reviewing polypharmacy and/or deprescribing is suboptimal. In our study, only one in four respondents thought that education in medical school had prepared them for deprescribing in clinical practice. Education is of utmost importance as it is one of the main facilitators of deprescribing as expressed in our study and also earlier by Irish pharmacists [35]. Recently, a review on geriatric medicine topics in medical curriculums [36] was published. The authors recommended integrating basic education on reviewing appropriate polypharmacy and deprescribing in the medical curriculum in a standardized way, because the worlds’ population is aging and every medical doctor should be prepared to effectively treat older patients with multiple morbidities, including deprescribing medications when appropriate. The lack of education is not surprising, because the geriatricians in our study were in medical school before deprescribing started to gain attention in the field of medicine. This is reflected in a significantly higher percentage of geriatricians-in-training who reported to have been educated or trained in reviewing polypharmacy and/or deprescribing in medical school compared to geriatricians (47 vs 23%, respectively). Apparently, deprescribing has recently gained attention in medical curricula. For geriatricians, judicious deprescribing can be regarded core business because they treat predominantly multimorbid older adults with disability, frailty, limited life expectancy, changed goals of care and polypharmacy. Therefore, advanced education and training should be provided in geriatric medicine training programs. In our study, however, only 72% of geriatricians and geriatricians-in-training had received education or training in reviewing polypharmacy and/or deprescribing. Of those, only 54% of geriatricians and 83% of geriatricians-in-training were educated/trained in geriatric medicine residency or fellowship.

In addition, deprescribing is complex due to scarcity of evidence-based recommendations with regard to the efficacy of medicines on the one hand, and the effects of deprescribing on the other hand in older, frail and multimorbid adults. Indeed, older adults are underrepresented in clinical medication trials [37], especially when they are frail and/or have comorbidities. As a result, disease-specific guidelines often lack recommendations on (de)prescribing medicines for these patients. Even scarcer are evidence-based guidelines specifically for managing polypharmacy and/or deprescribing in patients with disease clusters [38]. In our study, the lack of evidence-based (de)prescribing recommendations was among the most important barriers to deprescribing. We demonstrated that European geriatricians are likely to use guidelines or tools, even if these were published only recently (for example STOPPFall [39]). In fact, adherence to these guidelines and tools was one of the most reported reasons for deprescribing in our study. Therefore, in an effort to optimize future geriatricians (de)prescribing activities, it may be worthwhile to invest in clinical care guidelines that incorporate the best available evidence of both the effects of prescribing medications in multimorbid older adults, and the effects of deprescribing them.

Lastly, deprescribing may be challenging to geriatricians due to a contemporary lack of focus among patients and prescribers on the importance of deprescribing (potentially) inappropriate medications. Approximately 90% of respondents in our study believed a culture change is required for patients and prescribers, with an increasing focus on the possible benefits of deprescribing (potentially) inappropriate medications. Interventions targeting this culture change will likely benefit deprescribing.

Interestingly, the survey populations’ overall biggest barriers to deprescribing (systems and communication issues including lack of time, difficulty communicating with other doctors and patient reluctance) were not completely mirrored by the solutions participants thought would be most helpful to improve future deprescribing activities, e.g. educational resources. Although supportive educational resources such as guidelines and clinical decision support may be helpful to improve communication with other prescribers and patients and it may safe time, this does not solve systems issues where for example lack of resources may need to be addressed or the structuring of care pathways. When studied according to European region, however, the highest-ranked barrier and solution items did overlap largely, but some regional differences remained. To best fit the needs and wishes of geriatricians, and to improve their deprescribing activities, these differences should be addressed in future efforts to improve deprescribing.

### Strengths and limitations

Our survey has several strengths. This is the first study to comprehensively investigate deprescribing habits, attitudes, and needs of European geriatricians and geriatricians-in-training. We captured all phases of patient-centered deprescribing and the reasons for decision making with regard to deprescribing. We focused on physician-, patient- and healthcare system aspects, reflecting the fact that deprescribing decisions are influenced by complex interactions between clinical, social, and cultural factors [33]. Therefore, our results capture the deprescribing process in all its complexity. Uptake was widespread: approximately one in every 11 geriatricians in Europe participated (the estimated number of European geriatricians is 11,000), and nearly all EU (European Union) countries were represented in our study.

Our study also has several limitations. First, our results reflect geriatricians’ and geriatricians’-in-training beliefs regarding their deprescribing activities, and may diverge from their actual deprescribing activities in daily practice. For example, participants to our survey estimated they deprescribe medications they consider (potentially) inappropriate in 59% of patient consultations. Although quantitative data on geriatricians’ deprescribing efforts are scarce in the literature, this seems a relatively high percentage. In fact, even after visiting an experienced fall and syncope clinic, 91% of older patients experiencing falls or syncope remained on PIMs [40]. Recently, a deprescribing survey among GPs [41] revealed a difference between perceived practices and decisions made in hypothetical case-vignette scenarios. Another limitation is that we cannot rule out that the respondents who participated in the survey were more interested or had more expertise in deprescribing than their colleagues that did not participate. Consequently, generalizability may be negatively impacted. Lastly, the overall number of participants per country was too small to draw country-specific conclusions.

## Future perspectives

Our study increased our understanding of deprescribing habits and attitudes and the reasons for deprescribing (or not) among geriatricians and geriatricians-in-training across Europe, and identified regional differences herein. This knowledge is crucial for optimization of both the quality of (de)prescribing and the number of deprescribing activities, and implementation in clinical care. Based on the results of this study, the EuGMS SIG on Pharmacology will formulate and publish guidance for preferred deprescribing practices/approaches and education among European geriatricians. We will propose standards on how to address and succeed in deprescribing. To fit the needs of prescribers, and to meet the requirements for the safety and efficacy of deprescribing [42], our guidance will not be limited to the domain of the geriatrician, but will extend to patients, policy, and healthcare systems. As such, this study will contribute to the alignment and optimization of pharmacotherapy including deprescribing activities in older, multimorbid individuals.

## Conclusion

Geriatricians report several reasons for deprescribing but also important barriers and the lack of formal education. Based on our data, we recommend improving future deprescribing activities in multimorbid older individuals with polypharmacy by focusing on improved inter-professional communication/collaboration and shared decision-making, better education and evidence-based recommendations on (de)prescribing medicines to improve geriatricians’ future deprescribing activities.

## Supplementary Information

Below is the link to the electronic supplementary material.

Supplementary file1 (DOCX 290 KB)
