# Supplementary material for: Deprescribing practices, habits and attitudes of geriatricians and geriatricians-in-training across Europe: a large web-based survey
Source: Eur Geriatr Med. 2022 Nov 2;13(6):1455–66. doi: 10.1007/s41999-022-00702-9 (PMC9722796; doi:10.1007/s41999-022-00702-9)
Supplement: Supplementary file 1 — Supplementary file1 (DOCX 290 KB) [file 41999_2022_702_MOESM1_ESM.docx]

**Supplementary Table 1.** Number of geriatricians and geriatricians-in-training across the European regions participating in the survey.

**
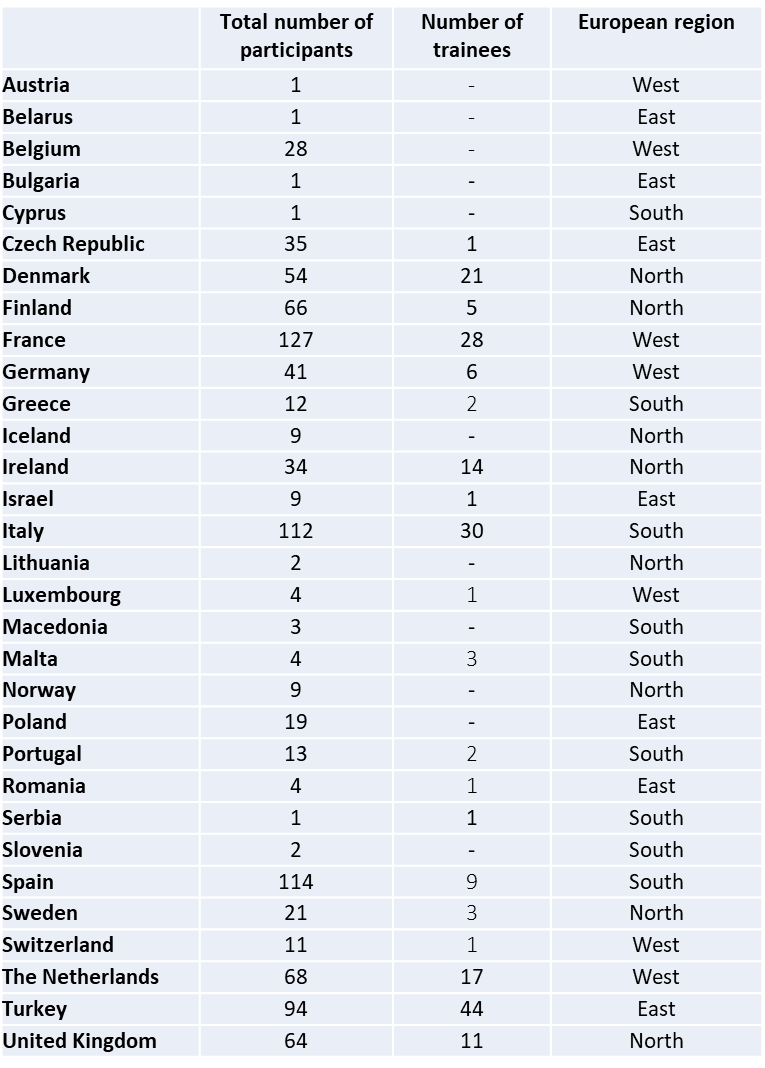
**

**Supplementary Figure 1.** Percentage of European geriatricians and geriatricians-in-training reporting to use deprescribing checklists and online deprescribing resources.

**Supplementary Table 2.** Education and training in reviewing polypharmacy and/or deprescribing among European geriatricians and geriatricians-in-training.

|  | **Total**  **(n=573)** | **Geriatricians**  **(n=458)** | **Geriatricians-in-training**  **(n=115)** |
| --- | --- | --- | --- |
| **Received education or training reviewing polypharmacy or deprescribing (% yes)** | 72 | 72 | 73 |
| **Setting of education or training (%)**  **-medical school**  **-residency and/or fellowship**  **-professional conferences or meetings**  **-other ^#^** | 28  60  71  12 | 23  54  75  58 | 47^*^  83 ^*^  53^*^  8 |
| **Hours of education or training in last 5 years (%)**  **<5**  **6-9**  **10-14**  **>15** | 27  32  17  25 | 26  32  17  25 | 30  32  15  24 |

^*^ Statistically significant difference (Chi square) at the 0.05 level.

^#^ Knowledge acquisition through e-learnings, literature search and/or online resources (for example Uptodate).

**Supplementary Figure 2.** Clinically most relevant barriers to deprescribing according to European geriatricians and geriatricians-in-training (in Quality Impact Indices).

On the X-axis, the QII (Quality Impact Index) score is shown, calculated by multiplying the score of the 4-point Likert scale to the statement “I find deprescribing challenging due to ... (=the respective factor)” (1=not challenging; 2=a little challenging; 3=challenging; 4=extremely challenging) by the fraction of participants that selected “yes” to the question “In the past month, have you been reluctant to deprescribe due to .... (=the respective factor)”; y-axis: 12 potential barriers to deprescribing.

**Supplementary Table 2.** The top 5 highest ranked reasons for deprescribing in multimorbid older adults according to geriatricians and geriatricians-in-training across European regions.


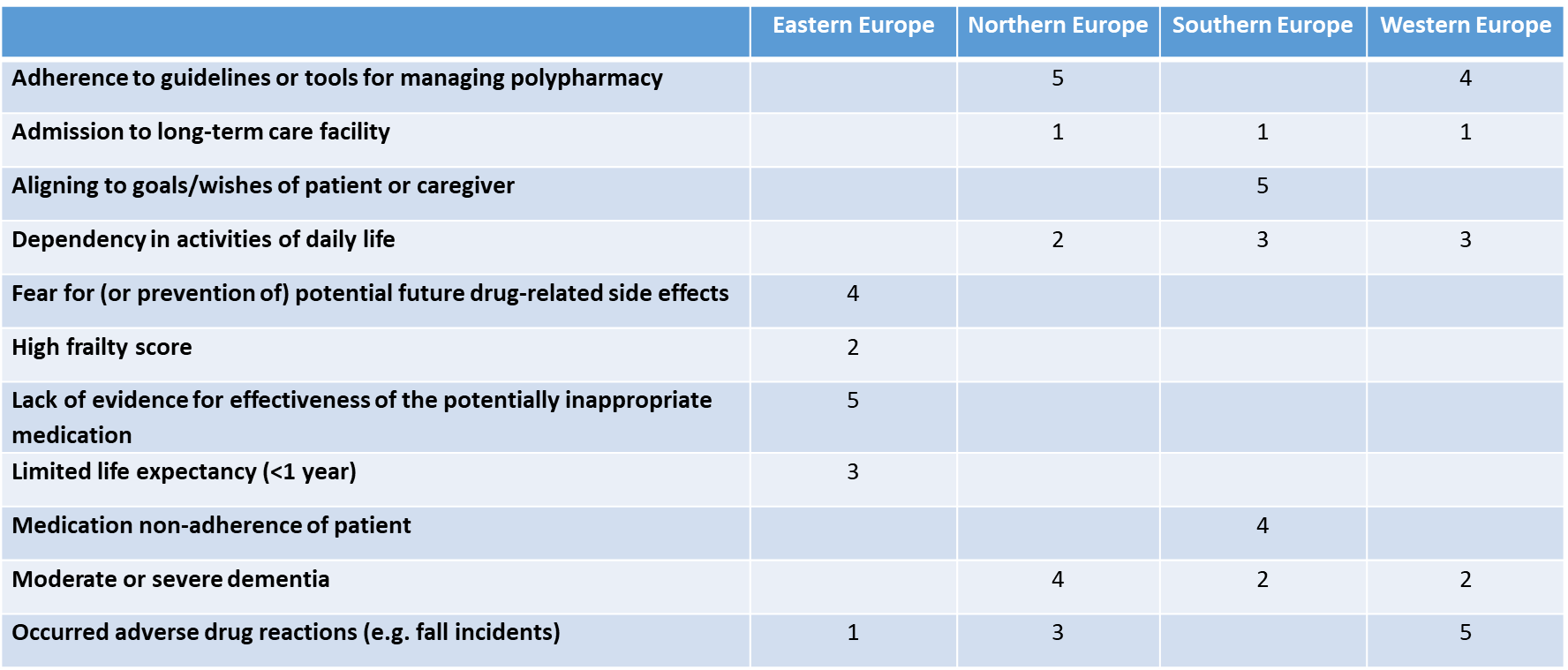


**Supplementary Table 3.** The top 10 highest ranked medications or medication groups that geriatricians and trainees across the European regions perceive having the highest risk of causing adverse drug reactions in multimorbid older adults.


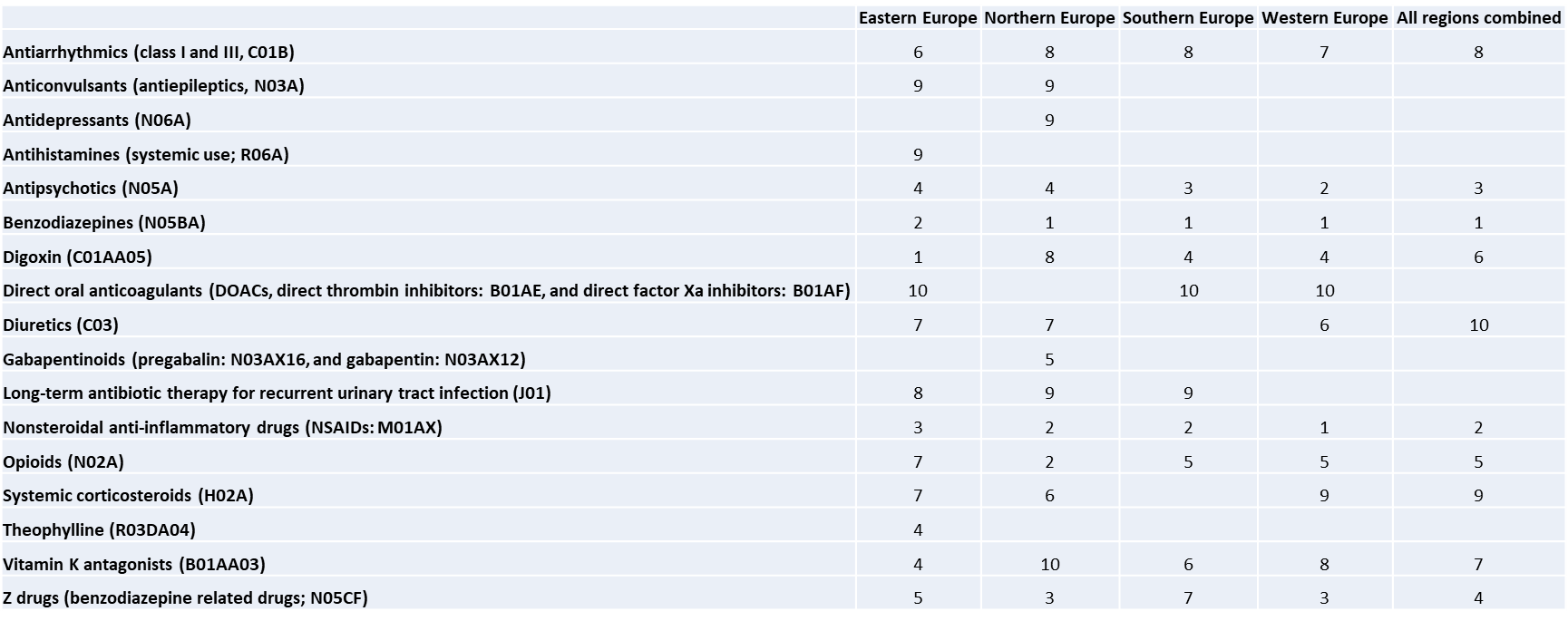


The total list consisted of 39 medications or medication groups. Participants were asked to report their agreement to the statement “In multimorbid older adults, I perceive the risk of adverse drug reactions related to this medication (group) as …” on a 5-point Likert scale (“very high” to “very low”). The number of participants selecting “very high” and “somewhat high” were calculated for analysis.

**Supplementary Table 4.** Ranking of the top 5 factors perceived as most clinically relevant barriers for deprescribing according to geriatricians and geriatricians-in-training across European regions.


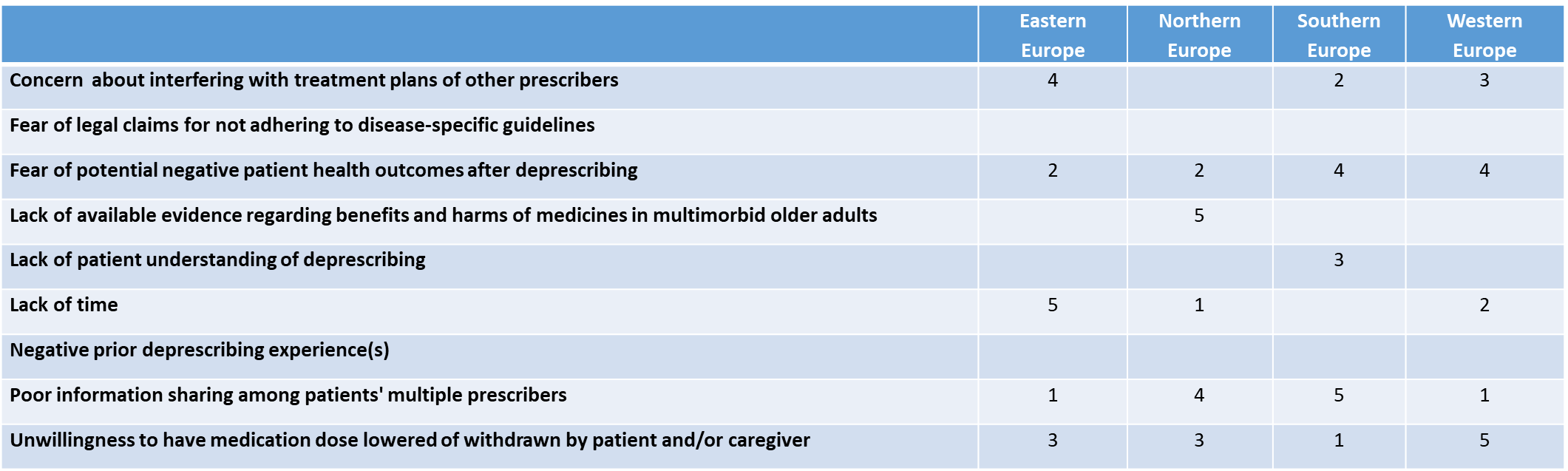


**Supplementary Figure 3.** Highest ranked factors geriatricians and trainees think would most likely increase their future deprescribing activities according to the different European regions (percentage of participants).

**EuGMS survey:**

**Geriatricians' deprescribing attitudes, practices and approaches across Europe**

Dear colleague,

Thank you for considering participation in this online survey.

Over recent decades, the number of medications prescribed to older adults has increased considerably due to increased prevalence of multimorbidities. While the benefits of these medications are evident, they have also contributed to inappropriate polypharmacy. Abundant evidence indicates that inappropriate polypharmacy leads to negative medical, economic and social consequences, such as adverse drug events and drug-related hospitalizations.

In this context, review of medications and deprescribing has emerged as an effective strategy. Deprescribing has been shown to reduce inappropriate polypharmacy and medication-related adverse events. However, the actual practice of deprescribing is not well-established yet. Health professionals still find it difficult to deprescribe for older multimorbid patients, as it is highly complex. In addition, agreed guidelines for deprescribing are limited across Europe.

We developed this online questionnaire in an effort to capture the needs of geriatricians across Europe and identify key areas of improvement for safe deprescribing. It includes questions on your current deprescribing practices, approaches and attitudes, deprescribing knowledge and needs/wishes for future deprescribing activities.

This survey will take approximately 20 minutes of your time and is approved by the Medical Ethical committee of the Academic Medical Center in Amsterdam. Your personal information and data will be processed anonymously and will not be distributed to third parties. Participation is voluntary.

Yours sincerely,

On behalf of the EuGMS SIG on Pharmacology,

Eveline van Poelgeest, MD PhD, internist-geriatrician and clinical pharmacologist

Lotta Seppala, MSc

Nathalie van der Velde, MD, PhD professor in geriatrics

There are 52 questions in this survey

*Informed Consent*

Before proceeding, please confirm that you have read the informed consent form above and tick the box if you agree with these terms.

Please choose only one of the following:


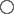
 I have read and accept the aforementioned and I will participate in this survey.

**Your Characteristics**

First, we would like to ask you some questions about you and your work environment.

**Q1. What is your age? Please indicate a numerical value (e.g. 40).**

**Q2. What is your sex? Please select one option.**


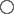
 Woman


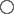
 Man


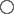
 Other


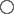
 Prefer not to say

**Q3. Which of the following best describes you at this moment? Please select one option.**

Please choose only one of the following:


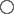
 Practicing geriatrician or specialist in care of older adults


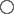
 Geriatrician or specialist in care of older adults *in training*


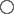
 Non-practicing geriatrician or specialist in care of older adults


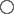
 Other, please specify.

If answer was 'Practicing geriatrician or specialist in care of older adults' *or* 'Geriatrician or specialist in care of older adults *in training*: **which of the following best describes you at this moment? Please select one option.**


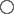
 General practice or community setting


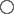
 Hospital, mostly outpatient clinic


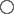
 Hospital, mostly clinical ward


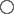
 Long-term care facility (e.g. nursing home) or rehabilitation setting


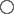
 Other, please specify.

**Q4. How many years of working experience as a doctor do you have?**

**Q5. How many years of working experience in care of geriatric patients do you have?**

**Q6. Do you have a (sub-) specialty (besides geriatrics)?**


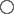
 Yes, please specify.


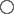
 No

**Q7. In which country are you currently working?**


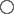
 Austria


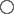
 Belgium


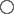
 Czech Republic


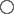
 Denmark


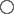
 Finland


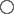
 France


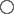
 Germany


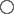
 Greece


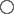
 Iceland


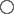
 Ireland


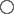
 Israel


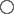
 Italy


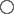
 Netherlands


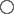
 Norway


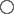
 Poland


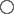
 Portugal


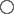
 Spain


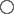
 Sweden


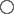
 Turkey


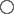
 United Kingdom


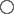
 Other, please specify.

**Q8. At your setting of practice, is there a dedicated deprescribing (outpatient) clinic available?**


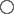
 Yes


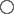
 No

If answer was “Yes”: **Which of the following healthcare specialists runs this clinic?**


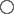
 Pharmacist


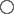
 Physician, clinical pharmacologist


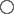
 Geriatrician


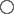
 Other, please specify.

And additionally: **Would you recommend a dedicated deprescribing clinic to your (inter-) national colleagues?**


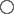
 Yes


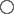
 No

**Q9. In your opinion, should the same health care professional be responsible for both selecting medicines to deprescribe and the follow-up monitoring phase?**


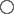
 Yes


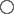
 No

If answer was “No”: Please specify who in your opinion should be responsible for selecting medicines to deprescribe and **Please specify who in your opinion should be responsible for the follow-up monitoring phase**.

**Your Deprescribing Approaches and Practices**

 Next, we would like to ask you about your deprescribing practices, approaches and behaviour. Please note that in this survey, deprescribing refers to planning, tapering and close monitoring during and after (inappropriate; see help box) medication withdrawal.

Help Box: Here, inappropriateness of medications can be based on individual patient characteristics/situations (e.g. no valid indication anymore) or based on specific criteria such as (e.g. the STOPP or Beers criteria).

**Q10. On average, how often do you deprescribe medications that you consider (potentially) inappropriate?**

% of consultations.

**Q11. Please answer the following statement: In multimorbid older adults, I perceive the risk of adverse drug reactions related to this medication (group) as …..**

|  | **Very low** | **Somewhat low** | **Neutral** | **Somewhat high** | **Very high** |
| --- | --- | --- | --- | --- | --- |
| **ACE inhibitors (C09A)** | 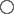 | 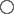 | 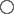 | 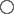 | 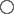 |
| **Angiotensin receptor blockers (C09CA)** | 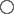 | 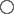 | 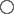 | 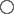 | 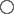 |
| **Antiarrhythmics (class I and III, C01B)** | 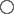 | 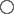 | 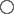 | 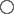 | 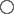 |
| **Antiplatelet drugs (B01AC)** | 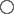 | 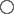 | 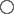 | 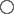 | 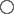 |
| **Beta blockers (C07)** | 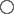 | 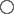 | 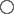 | 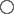 | 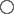 |
| **Calcium channel blockers (C08)** | 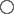 | 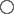 | 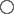 | 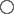 | 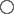 |
| **Digoxin (C01AA05)** | 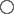 | 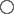 | 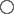 | 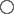 | 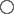 |
| **Direct oral anticoagulants (DOACs, direct thrombin inhibitors: B01AE, and direct factor Xa inhibitors: B01AF)** | 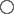 | 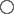 | 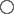 | 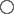 | 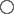 |
| **Diuretics (C03)** | 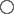 | 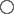 | 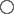 | 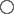 | 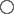 |
| **Lipid lowering medicines (C10A)** | 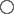 | 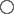 | 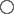 | 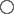 |  |
| **Nitrates (C01DA)** |  |  |  |  |  |
| **Vitamin K antagonists (B01AA03)** |  |  |  |  |  |
| **Anticonvulsants (antiepileptics, N03A)** |  |  |  |  |  |
| **Antidepressants (N06A)** |  |  |  |  |  |
| **Antipsychotics (N05A)** |  |  |  |  |  |
| **Antivertigo drugs (N07C)** |  |  |  |  |  |
| **Benzodiazepines (N05BA)** |  |  |  |  |  |
| **Cholinesterase inhibitors (N06DA)** |  |  |  |  |  |
| **Gabapentinoids (pregabalin: N03AX16, and gabapentin: N03AX12)** |  |  |  |  |  |
| **NMDA receptor antagonists (N06DX)** |  |  |  |  |  |
| **Z drugs (benzodiazepine related drugs; N05CF)** |  |  |  |  |  |
| **Bisphosphonates (M05BA)** |  |  |  |  |  |
| **Calcium supplements (A12A)** |  |  |  |  |  |
| **Estrogens (G03C)** |  |  |  |  |  |
| **Insulin (A10A)** |  |  |  |  |  |
| **Oral antidiabetics (A10B)** |  |  |  |  |  |
| **Systemic corticosteroids (H02A)** |  |  |  |  |  |
| **Thyroid hormones (H03AA)** |  |  |  |  |  |
| **Vitamin D (A11CC20)** |  |  |  |  |  |
| **Vitamins or supplements including iron supplements (A11), excluding vitamin D (A11CC20)** |  |  |  |  |  |
| **Acetaminophen (paracetamol; N02BE01)** |  |  |  |  |  |
| **Nonsteroidal anti-inflammatory drugs (NSAIDs: M01AX)** |  |  |  |  |  |
| **Opioids (N02A)** |  |  |  |  |  |
| **Antihistamines (systemic use; R06A)** |  |  |  |  |  |
| **Inhaled bronchodilators (R03A)** |  |  |  |  |  |
| **Long-term antibiotic therapy for recurrent urinary tract infection (J01)** |  |  |  |  |  |
| **Ophthalmic preparations (S01)** |  |  |  |  |  |
| **Proton pump inhibitors (A02BC) or H2 blockers (A02BA)** |  |  |  |  |  |
| **Theophylline (R03DA04)** |  |  |  |  |  |
| **Other, please specify.** |  | | | | |

**Q12. Please respond to the following statements.**

|  | **Yes** | **No** |
| --- | --- | --- |
| **In general, I am willing to deprescribe.** |  |  |
| **In general, I am proactive in reviewing medications and consider deprescribing potentially inappropriate medications in my patients.** |  |  |
| **In general, I take perceived risk of adverse drug reactions into account when deprescribing.** |  |  |
| **In general, I feel confident in deprescribing.** |  |  |
| **In general, I prefer deprescribing one medication at a time over deprescribing multiple medications at the same time.** |  |  |

**Q13. Please rate the following statements on deprescribing in older, multimorbid patients:**

|  | **Never** | **Rarely** | **Often** | **Always** |
| --- | --- | --- | --- | --- |
| **I collaborate with the GP when deprescribing.** |  |  |  |  |
| **I collaborate with a pharmacist or clinical pharmacologist when deprescribing.** |  |  |  |  |
| **I collaborate with other medical specialists (non GP or pharmacists) when deprescribing.** |  |  |  |  |
| **I collaborate with a specialized nurse when deprescribing.** |  |  |  |  |
| **Before deprescribing medications, I obtain a comprehensive medication history.** |  |  |  |  |
| **Before deprescribing medications, I estimate the risks and benefits of medications for the individual patient.** |  |  |  |  |
| **When reviewing medications, I take patient/caregiver preferences and goals into account.** |  |  |  |  |
| **I discuss deprescribing options with my patients and/or their caregivers.** |  |  |  |  |
| **I succeed in motivating patients and/or caregivers to deprescribe.** |  |  |  |  |
| **I take the final deprescribing decision myself.** |  |  |  |  |
| **I carefully plan initiation of medication withdrawal.** |  |  |  |  |
| **I document individualized monitoring plans during and after medication withdrawal.** |  |  |  |  |

If answer to “I collaborate with the GP when deprescribing” was 'Often' *or* 'Always': **How do you deprescribe medications initiated by GPs?**

 Directly myself

 After informing and consulting with the GP

 Other, please specify.

If answer to “I collaborate with a pharmacist or clinical pharmacologist when deprescribing” was 'Often' *or* 'Always': **In my opinion, pharmacists or clinical pharmacologists are important collaborators in deprescribing of potentially inappropriate medications in clinical practice.**

 Strongly disagree

 Disagree

 Neutral

 Agree

 Strongly agree

If answer to “I collaborate with other medical specialists (non GP or pharmacists) when deprescribing” was 'Often' *or* 'Always': **Which medical specialists do you collaborate with when deprescribing?**

 Cardiologist

 Psychiatrist

 Other, please specify.

And: **How do you deprescribe medications initiated by other treating medical specialists?**

 Directly myself

 After informing and consulting with the specialist

 Other, please specify.

If answer to “I collaborate with a specialized nurse when deprescribing” was 'Often' *or* 'Always': **In my opinion, specialized nurses are important collaborators in deprescribing of potentially inappropriate medications in clinical practice.**

 Strongly disagree

 Disagree

 Neutral

 Agree

 Strongly agree

**Your Education and Knowledge on Deprescribing**

Next, we would like to ask you questions regarding the education you received in deprescribing and the knowledge you obtained.

**Q14. Have you ever received education or training sessions on reviewing polypharmacy or deprescribing?**

 Yes

 No

If answer was “Yes”: **Where did you receive this education or training in deprescribing?** **Please choose all that apply.**

 Medical school

 Residency and/or fellowship

 Professional conferences or meetings

 Other, please specify.

And **Please estimate the number of hours of education or training regarding deprescribing you have received in the last 5 years.**

 Less than 5 hours

 5 to 10 hours

 10 to 15 hours

 Over 15 hours

And: **Education during my medical curriculum prepared me adequately for deprescribing in clinical practice.**

 Strongly disagree

 Disagree

 Neutral

 Agree

 Strongly Agree

**Q15. Do you use existing (inter-) national deprescribing or polypharmacy guidelines to review appropriateness of medication? Please select all that apply.**

 Yes, national guideline(s)

 Yes, international guideline(s)

 No

If answer is “Yes”: **Please specify.**

**Q16. Do you use any of the following deprescribing checklists, tools and resources? Please select all options that apply.**

 American Geriatrics Society Beers criteria

 Australian deprescribing recommendations (www.nswtag.org.au/deprescribing-tools)

 Canadian deprescribing algorithms (www.deprescribingnetwork.ca/algorithms)

 COME-ON

 CRIME criteria

 Deprescribing.org

 Drug burden index

 FORTA list

 Geriatric-Palliative algorithm

 GheoP3S tool

 LESS-CHRON

 Medstopper.com

 NORGEP-NH

 PRISCUS list

 Scottish Polypharmacy Guidance Realistic Prescribing

 STOPP START

 STOPPFalls

 STOPPFrail

 Tasmanian deprescribing recommendations (www.primaryhealthtas.com.au/resources/deprescribing-resources)

 TIME criteria

 Other, please specify.

 None of the above

**Q17. Please rate the following statements on deprescribing in older, multimorbid patients:**

|  | **Strongly disagree** | **Disagree** | **Neutral** | **Agree** | **Strongly agree** |
| --- | --- | --- | --- | --- | --- |
| **My general knowledge on deprescribing is good.** |  |  |  |  |  |
| **My knowledge on which drugs should not be stopped abruptly (but instead require stepwise tapering) is adequate.** |  |  |  |  |  |
| **In my opinion, a culture change is required for *prescribers*, increasing focus on the possible benefits of deprescribing (potentially) inappropriate medications.** |  |  |  |  |  |
| **In my opinion, a culture change is required for *patients*, increasing focus on the possible benefits of deprescribing (potentially) inappropriate medications.** |  |  |  |  |  |

**Your Barriers and Facilitators for Deprescribing**

Lastly, we would like to ask about the factors that facilitate you deprescribing and those that pose a challenge. In addition, we are interested in your needs/wishes for future deprescribing activities.

**Q18. What are your main reasons for considering deprescribing of (potentially) inappropriate medications? Please select the five options most important to you.**

 Acute hospital admission or emergency room visit

 Adherence to guidelines or tools for managing polypharmacy

 Admission to long-term care facility

 Aligning to goals/wishes of patient or caregiver

 Budgetary considerations

 Complaint of high pill burden

 Dependency in activities of daily life

 Fear for (or prevention of) potential future drug-related side effects

 High frailty score

 Lack of evidence for effectiveness of the potentially inappropriate medication

 Limited life expectancy (<= 1 year)

 Moderate or severe dementia

 Medication non-adherence of patient

 Occurred adverse drug reactions (e.g. fall incident)

 Patients' or caregivers' complaint of symptoms they relate to medication use and/or request to deprescribe

 Other, please specify.

**Q19. Please respond to the following statements.**

|  | **I find deprescribing challenging due to ...** | | | |  | **In the past month, have you been reluctant to deprescribe due to ...** | |
| --- | --- | --- | --- | --- | --- | --- | --- |
|  | **Not challenging** | **A little challenging** | **Challenging** | **Extremely challenging** |  | **Yes** | **No** |
| **Concern about interfering with treatment plans of other prescribers** |  |  |  |  |  |  |  |
| **Fear of legal claims for not adhering to disease-specific guidelines** |  |  |  |  |  |  |  |
| **Lack of available evidence regarding benefits and harms of medicines in multimorbid, older adults** |  |  |  |  |  |  |  |
| **Lack of available evidence regarding potential effects of deprescribing medicines in multimorbid, older adults** |  |  |  |  |  |  |  |
| **Fear of potential negative patient health outcomes after deprescribing (e.g. disease recurrence or withdrawal effects)** |  |  |  |  |  |  |  |
| **Lack of time** |  |  |  |  |  |  |  |
| **Lack of support** |  |  |  |  |  |  |  |
| **Lack of confidence in my deprescribing capabilities** |  |  |  |  |  |  |  |
| **Lack of patient understanding of deprescribing** |  |  |  |  |  |  |  |
| **Negative prior deprescribing experience(s)** |  |  |  |  |  |  |  |
| **Poor information sharing among patients' multiple prescribers (see help box)** |  |  |  |  |  |  |  |
| **Unwillingness to have medication dose lowered of withdrawn by patient and/or caregiver** |  |  |  |  |  |  |  |

Help Box regarding “Poor information sharing”: Example: no information regarding the reason for prescribing medications and effects on symptoms.

**Q20. Which of the following factors would most likely increase your deprescribing activities? Please select the 5 options that most apply to you.**
 A computerized clinical decision support system or app

 A national guideline containing deprescribing recommendations for medication (sub-) classes

 Availability of a dedicated specialized nurse to assist (e.g. in the monitoring phase)

 Better access to expert advice regarding pharmacotherapy

 Financial reimbursement (see help box)

 Improved information sharing between different prescribers

 Inclusion of practical advice on deprescribing regarding geriatric patients in "disease-specific" national guidelines

 Increased education and training in deprescribing

 Increased knowledge and skills in risk communication with patients and/or caregivers

 More detailed deprescribing recommendations for geriatric patients in national drug formularies/monographs

 More time

 Patients empowerment to deprescribing issues

 Other, Please specify.

Help Box regarding "Financial reimbursement": As an example, the Swedish government provided a financial reward to national regions that reduced potential inappropriate medication rates by a given percentage (reference: Factors influencing deprescribing for residents in Advanced Care Facilities: insights from General Practitioners in Australia and Sweden, BMC Family Practice, 2016).

If “Improved information sharing between different prescribers” was included: **What kind of information are you referring to?**

 Indication for medications

 Effect on symptom reduction after initiating medication

 Details on previous deprescribing attempts

 Adverse effects that may be related to the medication

 Effect of the medication withdrawal or continuation on quality of life

 Other, please specify.

Thank you for completing this survey.
